# Biocomputing: Beyond the Hype

**DOI:** 10.2196/100949

**Published:** 2026-05-28

**Authors:** Simon Spichak

**Keywords:** brain organoids, cloud computing, human-machine systems, drug discovery, cognition, brain mapping, artificial intelligence, bioethics

## Abstract

Biocomputing is a nascent but rapidly developing field at the intersection of biology and computer science. In this *News and Perspectives* article, JMIR Correspondent Simon Spichak reports on its current and potential applications for health care research and beyond.

**Key Takeaways:**

- Biocomputing connects human brain organoids to a computer to see how they respond to electrical and chemical stimuli.
- Some of the companies developing biocomputers allow for other researchers to access them via the cloud.
- In the near future, these systems could provide insights into brain and cognitive function and have applications in drug discovery, but some experts believe that biocomputers may have broader applications in the far future.

While venture capital is investing in data centers to bolster the computing power of artificial intelligence algorithms for research and other biomedical applications, scientists are turning to human brain cells as an alternative.

To start, scientists bathe human blood or skin cells in a cocktail of chemical factors to coax them back into a pluripotent (ie, capable of differentiating into almost any other cell type) stem cell state. Over the course of a year, these cells differentiate and mature into small spheres of brain tissue called organoids. Growing them on top of multielectrode arrays in a hardware shell has allowed research groups to stimulate and measure how these organoids respond to electrical and chemical signals. Cortical Labs out of Australia and FinalSpark out of Switzerland are leading the way, allowing other scientists to purchase the hardware to set up a biocomputer or access these platforms through the cloud.

While the field is still in the nascent stages, researchers believe biocomputing could help neuroscientists better understand how the brain works, play a role in drug development, and perhaps provide an energy-efficient alternative to AI computing.

## Why Build Biocomputers?

When Fred Jordan, PhD, and Martin Kutter, PhD, founded FinalSpark, they wanted to build a thinking machine using artificial neural networks (ANNs)—simple mathematical models of neurons. But ANNs, which would become the backbone of large language models, weren’t providing the progress they hoped, while also consuming substantial amounts of energy.

Despite requiring careful care and upkeep, these biocomputers are still more efficient than any supercomputer. While exact measures are scarce, an analysis done on rat brains suggests the brain consumes 30 watts of power a day, less than some lightbulbs. “Maybe it’s a better idea to use real neurons,” said Jordan.

Unlike artificial intelligence, Brett Kagan, PhD, the chief scientific officer of Cortical Labs, says that biological networks are better at dealing with chaotic, noisy data and can learn with substantially less input.

“The complexity of how biological neural systems compute and process information is a huge question,” said Kagan. “But what we’re doing is we’re able to break it down now to the level of information physics.”

**Figure:**
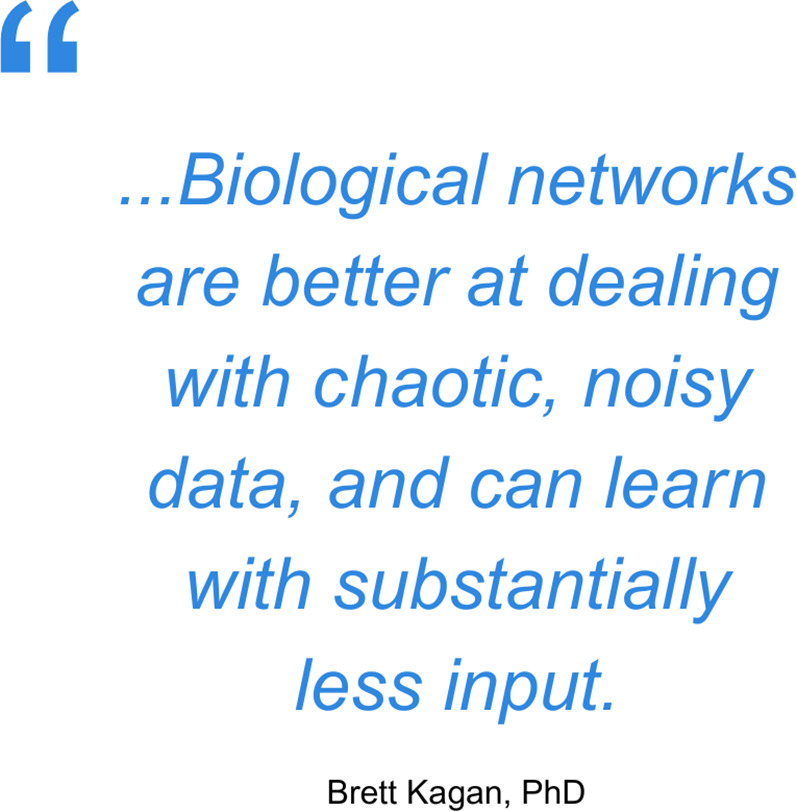


## Bioethics and Potential Risks

Bioethicists have taken a keen interest in the field as well.

“We are so far away from the experimental side to something which is a relevant material for ethicists,” said Thomas Hartung, MD, PhD, a professor at Johns Hopkins who works in the biocomputing field. His lab takes an embedded ethics approach, where an ethicist sits in on all of their laboratory meetings so that they can have discussions before any problems arise around privacy or consciousness within these systems.

Cortical Labs and FinalSpark are actively working with bioethicists to ensure that their biocomputers are used responsibly. The brain organoids used for biocomputing raise similar concerns to stem cell and organoid research, including the moral status and development of potential consciousness in more advanced models, informed consent from donors, and issues around commercialization, ownership, and patents.

## What Are Biocomputers Used For?

By 2023, FinalSpark became the first biocomputing lab to provide remote access to biocomputing for research. Their hardware connects brain organoids to an interface that allows for chemical and electrical stimulation.

“I give you access to my brain organoids,” said Jordan. “Then you can write Python code, and I will give you an API to stimulate the neurons and to listen to them.” FinalSpark gave 9 universities free access to their biocomputer, Neuroplatform, and provides access to additional researchers.

Some of the work focuses on understanding how the connectome—or detailed map of neural connections—changes in response to brain stimulation. They’ve also connected the Neuroplatform to a large language model that can directly prompt the system and run experiments for researchers. “The quality of the research was similar to what would have been done with the same tools,” said Jordan.

Cortical Labs, meanwhile, has captivated headlines by training their biocomputers to play video games. In 2022, its scientists “taught” a 2D brain organoid connected to a computer how to play Pong. They created a simulated environment where aspects of the video game, like the location of the balls and paddles, were transduced into electrical signals.

With Pong, Kagan recalled, the team had to develop everything from the ground up. Since then, the company has created a hardware system called the CL1, as well as software, that allows anyone to use the biocomputer. Earlier this year, a graduate student programmed Cortical Labs’ biocomputing platform to play the 1993 first-person shooter game *DOOM* during a hackathon. *DOOM* is a “nice demonstration” of what people with little experience can build within 7-10 days.

“You can actually allow anyone to build amazing things with this technology,” said Kagan. “Not just the scientists in the lab who have spent their life doing it.”

While these companies aren’t trying to rebuild the brain, using its component cells could provide a leg up in computing. Both companies now allow people to tap into the cloud to use the compute power of brain cells grown in a dish.

The company currently offers three ways for researchers and other companies to use the CL1. They can buy the hardware, while growing the cells and writing their own software to analyze them. They can access the CL1 through the cloud and interact with the cells remotely. Or they can have Cortical Labs run the entire experiment themselves.

Hartung believes that while these cloud approaches offer the opportunity for scientists to familiarize themselves with the systems, he thinks that systematic experiments still need to be run in person.

These biocomputing platforms are already in use for drug discovery, allowing scientists to test how different experimental medications might affect how the organoids learn. It also provides a glimpse into the inner workings of the brain.

## The Future of Biocomputing

The field is still in its nascent era. Kagan is optimistic about research groups emerging in Europe, China, Japan, and the United States.

Jordan sees mastering training as the biggest challenge for biocomputing. In the 1990s, he said, scientists puzzled this out for ANNs, allowing them to modify the weight of connections to improve the accuracy of the output.

With an ANN, the weights don’t change unless they’re reprogrammed. But brain organoids are still actively firing off even without any external input and the system is far more complicated. Breaking this code would allow scientists to master these networks.

Hartung said that a biocomputing system that learns might take several years of training, not unlike human learning, which will limit its applications. He believes it could help engineers develop neuromorphic computing—hardware that better mimics the human brain.

But Jordan and Kagan are more optimistic. “I think in 10 years,” said Jordan, “biocomputers will be way more useful than quantum computers today.”

